# Post-Mortem diagnosis of dementia by informant interview

**DOI:** 10.1590/S1980-57642010DN40200011

**Published:** 2010

**Authors:** Renata Eloah de Lucena Ferretti, Antonio Eduardo Damin, Sonia Maria Dozzi Brucki, Lilian Schafirovits Morillo, Tibor Rilho Perroco, Flávia Campora, Eliza Guccione Moreira, Érika Silvério Balbino, Maria do Carmo de Ascenção Lima, Camila Battela, Lumena Ruiz, Lea Tenenholz Grinberg, José Marcelo Farfel, Renata Elaine Paraiso Leite, Claudia Kimie Suemoto, Carlos Augusto Pasqualucci, Sérgio Rosemberg, Paulo Hilário Nascimento Saldiva, Wilson Jacob-Filho, Ricardo Nitrini

**Affiliations:** 1 Division of Geriatrics, University of São Paulo Medical School, São Paulo SP, Brazil.; 2 Brazilian Brain Bank of the Aging Brain Study Group - Laboratory of Medical Investigations 22 (LIM 22).; 3 University of ABC.; 4 RCCD, Reference Center for Cognitive Disorders, University of São Paulo Medical School, São Paulo SP, Brazil.; 5 Department of Neurology, University of São Paulo Medical School, São Paulo SP, Brazil.; 6 Department of Psychiatry,University of São Paulo Medical School, São Paulo SP, Brazil.; 7 Department of Pathology, University of São Paulo Medical School, São Paulo SP, Brazil.; 8 Department of Neurology, University of California, San Francisco.; 9 São Paulo Autopsy Service.

**Keywords:** brain bank, postmortem diagnosis, dementia, aging, informant-interview

## Abstract

The diagnosis of normal cognition or dementia in the Brazilian Brain Bank of the Aging Brain Study Group (BBBABSG) has relied on postmortem interview with an informant.

**Objectives:**

To ascertain the sensitivity and specificity of postmortem diagnosis based on informant interview compared against the diagnosis established at a memory clinic.

**Methods:**

A prospective study was conducted at the BBBABSG and at the Reference Center for Cognitive Disorders (RCCD), a specialized memory clinic of the Hospital das Clínicas, University of São Paulo Medical School. Control subjects and cognitively impaired subjects were referred from the Hospital das Clínicas to the RCCD where subjects and their informants were assessed. The same informant was then interviewed at the BBBABSG. Specialists’ panel consensus, in each group, determined the final diagnosis of the case, blind to other center’s diagnosis. Data was compared for frequency of diagnostic equivalence. For this study, the diagnosis established at the RCCD was accepted as the gold standard. Sensitivity and specificity were computed.

**Results:**

Ninety individuals were included, 45 with dementia and 45 without dementia (26 cognitively normal and 19 cognitively impaired but non-demented). The informant interview at the BBBABSG had a sensitivity of 86.6% and specificity of 84.4% for the diagnosis of dementia, and a sensitivity of 65.3% and specificity of 93.7% for the diagnosis of normal cognition.

**Conclusions:**

The informant interview used at the BBBABSG has a high specificity and sensitivity for the diagnosis of dementia as well as a high specificity for the diagnosis of normal cognition.

## Abstract (pt)

Os diagnósticos de cognição normal ou de demência dos casos do Banco de Encéfalos do Grupo Brasileiro de Estudos de Envelhecimento Cerebral tem se baseado em entrevista realizada com informante.

**Objetivos:**

Verificar a sensibilidade e especificidade do diagnóstico postmortem baseado em entrevista com informante quando comparado com o diagnóstico estabelecido em clínica de memória.

**Métodos:**

Um estudo prospectivo foi conduzido no Banco de Encéfalos e no Centro de Referência em Distúrbios Cognitivos (CEREDIC), uma clínica especializada do Hospital das Clínicas da Faculdade de Medicina da Universidade de São Paulo. Indivíduos controle e cognitivamente comprometidos foram encaminhados pelo Hospital das Clínicas ao CEREDIC onde os indivíduos foram avaliados e os informantes entrevistados. O mesmo informante foi então entrevistado pela equipe do Banco de Encéfalos. Consenso em painel de especialistas, em cada centro, estabeleceu o diagnóstico final em cada caso, sem conhecimento do diagnóstico do outro centro. Os diagnósticos foram comparados, admitindo-se o diagnóstico estabelecido no CEREDIC como padrão-ouro. Sensibilidade e especificidade foram calculadas.

**Resultados:**

90 indivíduos foram incluídos, 45 com demência e 45 não-dementes (26 cognitivamente normais e 19 com comprometimento cognitivo sem demência). A entrevista realizada no Banco de Encéfalos teve sensibilidade de 86,6% e especificidade de 84,4% para o diagnóstico de demência e sensibilidade de 65,3% e especificidade de 93,7% para o diagnóstico de cognição normal.

**Conclusões:**

A entrevista com informante realizada no Banco de Encéfalos do Grupo Brasileiro de Estudos de Envelhecimento Cerebral tem altas sensibilidade e especificidade para o diagnóstico de demência e alta especificidade para o diagnóstico de cognição normal.

The study of the morphological and biological changes occurring in the brain in normal aging and in dementia comprise one of the most challenging frontiers in neuroscience. In order to accomplish the objectives of investigating brain of demented and non-demented elderly, brain banks have been implemented in many countries.^1,2^ As there has been a sharp decline in autopsy rates worldwide in recent decades, it has been difficult for the brain banks to collect significant brain samples, especially from normal volunteers.^3^

Brain banks depend on the donation of the brain by patients or their family or by healthy individuals. Usually, brain banks are linked to memory clinics where patients with dementia or cognitive impairment are followed with sequential neuropsychological evaluations until death. However, even with careful follow-up, there may be long intervals between the last evaluation and death. This also holds true for the cognitively normal volunteer, who may have converted to mild cognitive impairment or to dementia between last evaluation and death.^4,5^

In São Paulo, Brazil, a city with approximately 11 million inhabitants, autopsies are compulsory for those dying without an established cause of death, and all autopsies of natural deaths occurring in the area are performed in the São Paulo Autopsy Service (SPAS) by a medically qualified pathologist assisted by nationally certified technicians. In 2004, the Brain Bank of the Brazilian Aging Brain Study Group (BBBABSG) was founded and located adjacent to the SPAS facilities. In the BBBABSG, brains from deceased subjects aged 50 years or older are collected whenever it is possible to obtain collateral-source information on the past medical history of the deceased from a reliable informant. The possibility of obtaining a large number of brains from the SPAS, including brains from non-demented individuals, was confirmed when 1601 brains were collected in the first 21 months of activities of the BBBABSG.^5^

However, the clinical diagnosis of normal cognition or dementia established in the BBBABSG was based exclusively on the post-mortem interview with an informant. The reliability of this diagnosis in our bank had not been ascertained and was a critical aspect for our present and future studies.

The value of using questionnaires for the diagnosis of dementia in clinical practice and epidemiological studies have already been confirmed.^6,7^ A few previous studies have also evaluated the accuracy of post-mortem diagnosis of dementia using questionnaires and retrospective interview with an informant, and reported high sensitivity and specificity for the diagnosis of dementia or Alzheimer’s disease (AD).^8-11^

The objective of this study was to determine the sensitivity and specificity of the postmortem diagnoses based on the informant interview compared against the diagnosis established at a memory clinic, assumed as the gold-standard diagnosis for this study.

## Methods

Brain donations to the BBBABSG are made by the Next of Kin (NOK) of individuals who die in the metropolitan area of São Paulo and are taken to the São Paulo Autopsy Service (SPAS). At the time corpses arrive at the SPAS the NOKs are informed about the possibility of donating their deceased family member’s brain for study and research proposes.

After written informed consent, the NOK is interviewed by a team of baccalaureate nurse undergraduate students, supervised by a gerontologist nurse who aims to gather all relevant information to determine the clinical, functional and cognitive status of the case. This interview takes around 40 minutes.

The complete methodology of the BBBABSG has been described elsewhere^5,12^ and was approved by the local Research Ethics Committee. Ethical aspects follow the Brazilian requirements, which are based on international standards.

### BBBABSG clinical and functional assessments

**Figure 1:**
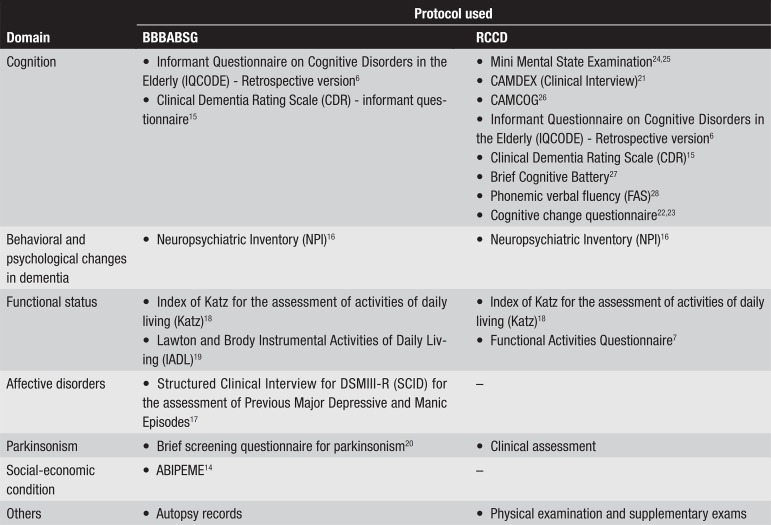
Protocols used by the Brazilian Brain Bank of the Aging Brain Study Group (BBBABSG) and Reference Center for Cognitive Disorders (RCCD), for clinical and functional assessment. CAMCOG, Cambridge Cognitive Examination; CAMDEX, The Cambridge Mental Disorders of the Elderly Examination.

The protocol used by the bank to gather information consists of a semi-structured retrospective questionnaire, composed by validated scales that cover major functional abilities, as depicted in Figure 1. Part one of the instrument gathers demographic data, and a complete anamnesis supplies information about the whole medical history of the subject. Autopsy records are also assessed for cause of death and associated causes.

The diagnosis of each case is derived from the semi-structured interview and a consensus panel between the gerontologist nurse and one neurologist with expertise in dementia, where all information obtained at the interview is taken into account, aiming to reach the best estimated diagnosis.

### The Reference Center in Cognitive Disorders (RCCD)

This center is an outpatient university-associated memory clinic, linked to the Hospital das Clínicas of the University of São Paulo (HC-USP) and is dedicated to the healthcare assistance of adult and elderly patients suffering from cognitive disorders. Patients and their caregivers receive integral diagnostic and therapeutic assistance, through a multi and interdisciplinary team.

The RCCD is a specialized center where every patient with cognitive disorders is seen by a team formed by a neurologist, geriatrician and psychiatrist and also by a multidisciplinary team including a psychologist, speech pathologist and occupational therapist. In the RCCD, the patient is submitted to a clinical interview together with a reliable informant, usually a family member. The patient is then evaluated with neuropsychological tests while the informant answers questionnaires, both shown in Figure 1. After the clinical exam, the patient undergoes neuropsychological and/or language evaluations, and also laboratory and neuroimaging studies as proposed by the Brazilian Academy of Neurology.^13^ All data are taken into account to reach the diagnosis in the consensus meeting.

### Study design

**Figure 2:**
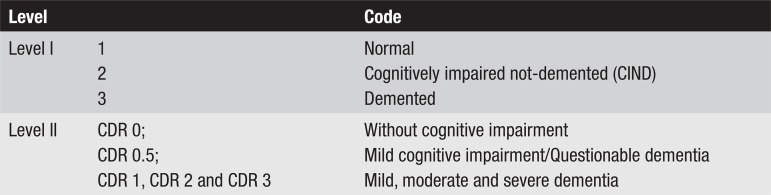
Levels of comparison at which diagnosis were defined.

A prospective study was performed in which normal controls and cognitively impaired subjects, together with informants (or caregivers), were referred from the HC-USP to the RCCD. The study was designed to reproduce the exact methodology of the BBBABSG. Figure 2 illustrates the flowchart for the study.

Cases were sourced from two different outpatient clinics belonging to HC-USP namely, Geriatrics and Neurology outpatient clinics. At these centers, both normal control and cognitively impaired subjects with their caregivers or informants were invited to take part in the study and if accepted, were then referred to the RCCD.

Patients and their caregivers were first assessed at the RCCD group, after signing a written informed consent. The complete battery was filled in. Subsequently, only the caregivers or informants were assessed at the BBBABSG, which rated each case based solely on the assessment of data provided by informant’s responses. Informants were instructed not to tell the BBBABSG if the patient had a diagnosis of dementia or what medications had been prescribed.

Both assessments used their original protocols and the RCCD diagnosis was assumed as the gold-standard for this study. None of the groups knew the origin of the cases to avoid bias in classification.

Specialists’ panel consensus, in each group, determined the final diagnosis of each case. Since the BBBABSG did not have access to patients and supplementary exams, the consensus panel used the methodology approach for “Best Estimated Diagnosis”. Investigators rated each case for the presence or absence of cognitive impairment and were blinded to the other group’s final diagnosis.

The diagnoses were classified into three levels at both centers. The first level classified the subjects into normal, cognitively impaired not-demented (CIND), and demented. The second level classified the individuals using the Clinical Dementia Rating (CDR) scale,^15^ using the complete version at the RCCD and the informant-only version at the BBBABSG. The third level was the final or the diagnosis of the disease causing dementia. For this article, only the first two levels shall be analyzed. At level I a cognitively impaired not-demented (CIND) diagnosis was established in subjects that presented some cognitive impairment compared to normal controls of the same age and education, but with preserved functional activities.

Levels of comparison were created aiming to standardize diagnosis equivalence in both groups, as shown in Figure 2.

### Case stratification and analysis procedures

The sample was stratified into two groups, BBBABSG and RCCD, and again into two subgroups according to level of diagnosis. Data was compared for frequency of diagnostic equivalence at each level. Sensitivity and specificity were computed.

For descriptive statistics the Statistical Package for the Social Sciences, version 14.0, was used. Sensitivity and specificity of the diagnosis obtained at the BBBABSG was obtained by comparing with the gold-standard diagnosis of the RCCD.

This study was approved by the Research Ethics Committee of the HC-USP, Brazil.

## Results

Ninety individuals (47 women) with mean age of 69.9±8.7 years old, ranging from 50 to 87y, and mean schooling of 5.7±4.3 years, ranging from 0 to 17y, were included in this study. The majority of the individuals were Caucasian (72.2%). Table 1 shows the classifications according to the complete evaluation performed by RCCD and based on the informant interview performed by the BBBABSG.

**Table 1:** Diagnoses of the individuals by the Reference Center for Cognitive Disorders (RCCD) and by the Brain Bank of the Brazilian Aging Brain Study Group (BBBABSG).

BBBABSG	RCCD			
	Normal	CIND	Dementia	Total
Normal	17	2	2	21
CIND	8	11	4	23
Dementia	1	6	39	46
Total	26	19	45	90

CIND, cognitively impaired not-demented.

There were 45 individuals with dementia and 45 non-demented (26 cognitively normal and 19 CIND). The sensitivity and specificity of the dementia diagnosis (versus no dementia) was 86.6% and 84.4%, respectively.

For the diagnosis of CIND, the sensitivity of the informant interview was 57.8% and the specificity, 83.0%. The sensitivity of the diagnosis of normal (for cognitively normal) by the informant interview was 65.3% and the specificity was 93.7%.

Analysis of the classifications according to the CDR revealed that only two patients with CDR 0 were classified as CDR > 0.5 (i.e., with dementia) by the informant interview (Table 2). Moreover, all patients with CDR 2 or 3 were classified as demented (CDR≥1).

**Table 2:** Classifications according to the CDR scale by the Reference Center for Cognitive Disorders (RCCD) and by the Brain Bank of the Brazilian Aging Brain Study Group (BBBABSG).

BBBABSG	RCCD					
	CDR 0	CDR 0.5	CDR 1	CDR 2	CDR 3	Total
CDR 0	16	2	2	-	-	20
CDR 0.5	8	11	5	-	-	24
CDR 1	2	4	15	2	-	23
CDR 2	-		10	8	1	19
CDR 3	-	-	3	-	1	4
Total	26	17	35	10	2	90

The overall agreement observed for both forms of CDR application was 56.6%.

When the diagnosis of normal cognition by the BBBABSG was established by combining the CDR 0 and IQCODE score < 3.42, a procedure used in several as yet unpublished studies, sensitivity was 61.5% and specificity was 94.5% for the diagnosis of normal cognition.

## Discussion

Both the sensitivity and specificity of the informant interview used at the BBBABSG for the diagnosis of dementia were high, at approximately 85%.

For the diagnosis of normal cognition the sensitivity was 65.3%, and was even lower for the diagnosis of CIND. This may be explained by the difficulty in separating normal cognition from CIND without cognitive tests, because memory complaints are very frequent in the elderly, even when corroborated by informants. By contrast, the specificity of the diagnosis of normal cognition was high, an important finding because it lends weight to the diagnosis of “normal” or “control” individuals of the BBBABSG. When the IQCODE was combined with the CDR, specificity for the diagnosis of normal cognition was even higher.

The value of questionnaires for the diagnosis of dementia has been confirmed by several studies. According to Jorm AF et al., the sensitivity and specificity of the IQCODE were 82.5% and 73%, respectively, when the DSM-III-R criteria were used.^6^ The Functional Activities Questionnaire also attained high sensitivity (85%) and specificity (81%) in distinguishing between normal and demented individuals.^7^ The Cognitive Change Questionnaire also attained high accuracy in this differentiation, even when individuals with questionable dementia were included in the sample of normal and demented individuals.^22^ Other studies have corroborated the value of questionnaires and the combination of questionnaires with cognitive tests for the diagnosis of dementia.^29-32^

The postmortem diagnosis of dementia has been evaluated by a few studies in the literature. Kukul and Larson (1989)^8^ used a questionnaire including the DSM-III criteria for primary degenerative dementia in conjunction with the Hachinski Ischemic Scale and reported high sensitivity but low specificity for the diagnosis of primary degenerative dementia. Davis et al. (1991)^9^ used a structured telephone interview and reported very high sensitivity and specificity (100% for both) for the diagnosis of dementia in 27 cases. Rockwood et al. (1998)^10^ also reported a high accuracy for the retrospective diagnosis of dementia using a semi-structured interview, although most of their cases had diagnoses of severe dementia. Ellis et al. (1998)^11^ compared the diagnoses obtained through a postmortem structured telephone interview against both antemortem clinical diagnoses and neuropathological diagnoses. For both comparisons, the postmortem interview showed high sensitivity and specificity. Our study reinforces the value of the postmortem interview for the diagnosis of dementia described by these previous studies.

The CDR is an important tool for the diagnosis of dementia and for grading its severity. The overall agreement between CDR scores obtained exclusively through the interview (“informant CDR”) and those on the complete CDR (“clinician CDR”) was not high in our study (56.6%). Waite et al. (1999)^33^ compared both these forms of the CDR in 360 elderly evaluated in a community survey and found moderate agreement whereas Davies et al. (1991)^9^ found high agreement between antemortem and postmortem CDR.

There are limitations to our study, principally because we were unable to fully replicate the conditions of the interview at the BBABSG. Firstly, although all questionnaires used in the informant interview of the BBBABSG were used in this study, the real situation of a postmortem interview could not be reproduced in this study. It is possible that the informant’s emotional state could have an impact on the quality of the information. Davis et al. (1991)^9^ reported that when they compared the CDR obtained at the time of the subject’s death both with the antemortem CDR and with the CDR obtained through an interview conducted 2 to 21 months later, the agreement was high. However, the agreement between the CDR obtained at the time of death with antemortem CDR was 85.7%, while the agreement between the later CDR and antemortem CDR was 70%. This finding reinforces the use of the immediate postmortem interview we conduct at the BBBABSG, but future studies by our group should compare information obtained at the postmortem interview with a later interview.

Another limitation constituted the high frequency of dementia in the individuals included in this study. Forty-five subjects of the total sample were demented, a much higher prevalence than that found in the deceased individuals of the BBBABSG. Ideally, a prevalence of dementia of 15 to 20% of the individuals would have better replicated the usual prevalence of dementia in the BBBABSG.

In spite of these limitations it is possible to conclude that the informant interview used at the BBBABSG has high specificity and sensitivity for the diagnosis of dementia and high specificity for the diagnosis of normal cognition.
